# Survival states as indicators of learning performance and biological stress in refugee children: a cross-sectional study with a comparison group

**DOI:** 10.1186/s12888-021-03233-y

**Published:** 2021-05-03

**Authors:** Andrea Hahnefeld, Thorsten Sukale, Elena Weigand, Katharina Münch, Sigrid Aberl, Lea V. Eckler, Davin Schmidt, Anna Friedmann, Paul L. Plener, Jörg M. Fegert, Volker Mall

**Affiliations:** 1 Chair of Social Pediatrics, TUM School of Medicine, Technical University of Munich, kbo Kinderzentrum, Heiglhofstrasse 65, 81377 Munich, Germany; 2 kbo Kinderzentrum, Heiglhofstr. 65, 81377 Munich, Germany; 3 Department of Child and Adolescent Psychiatry and Psychotherapy, University of Ulm, Steinhövelstraße 5, 89075 Ulm, Germany; 4 Department of Psychosomatic Medicine and Psychotherapy, Technical University of Munich, Ismaninger Straße 22, 81675 Munich, Germany; 5 Munich Municipal Hospital Group, Department of Child and Adolescent Psychosomatic Medicine, Kölner Platz 1, 80804 Munich, Germany; 6 Department of Child and Adolescent Psychiatry, Medical University of Vienna, Währingergürtel 18-20, 1090 Wien, Austria

**Keywords:** Refugee, Children, PTSD, Cortisol, Stress

## Abstract

**Background:**

Our goal was to accurately detect young children at risk for long-term psychiatric disturbances after potentially traumatic experiences in the course of relocation. In addition to detailed assessment of parent-rated parent and child symptomatology, we focused on disruptive behaviors in the education environment summarized as survival states, as these frequently lead to clinical referral.

**Methods:**

We screened 52 refugee children aged 3–7 (M = 5.14 years, SD = 1.17) for symptoms of Posttraumatic Stress Disorder (PTSD) with the Child and Adolescent Trauma Screening (CATS) in parent rating. The parents’ mental health was assessed using the Refugee Health Screener (RHS-15). Furthermore, the child’s educators were asked to evaluate the pathological survival states of the child and we made a general assessment of the children’s symptoms with the Strengths and Difficulties Questionnaire (SDQ) rated by parents and educators. Children in the refugee sample completed a working memory learning task (Subtest Atlantis from the Kaufmann Assessment Battery for Children, KABC-II) and delivered saliva samples for testing of the cortisol level.

**Results:**

The parental rating of their child’s PTSD symptoms was significantly related to their own mental well-being (*r* = .50, *p* < .001). Children with survival states in educator ratings exhibited weaker learning performance (*F* = 3.49, *p* < .05) and higher evening cortisol levels (U = 113, z = − 1.7, *p* < .05, one-tailed).

**Conclusions:**

Survival states are promising indicators for children’s learning performance and distress level complementary to parent rating of child PTSD, which is highly intercorrelated with the parents’ own symptom load.

**Trial registration:**

*Trial registration number*: DRKS00021150 on DRKS

*Date of registration*: 04.08.2020 retrospectively registered

## Background

During the recent large-scale population movement, estimations for people forcibly being displaced from their homes or escaping conflict and persecution globally rose to 68.5 million [1], with minors comprising about half of all refugees [2]. Likewise, there are increasingly more young children among asylum seekers in Germany. In 2019, children 10 years of age and younger made up 40% of the 142.509 refugees arriving in Germany [3].

The reasons and circumstances for their flight are often associated with potentially traumatic experiences, both personal and familial [4, 5], for example war, violence, separation and long routes with repeated relocation from conflict areas [6]. Children are suggested to be especially vulnerable to these cumulative psychosocial stress factors in the sense of being continuously exposed to an unpredictable environment [4, 7–9]. Estimates from previous studies on PTSD levels in refugee children samples who had resettled in Western countries show increased prevalence rates between 19 and 54% [4, 10–12] compared to 15.9% among trauma exposed children and adolescents in the general population [13]. In our preceding study with Syrian refugees in a German camp 26% of the 0–6 -year-old children and 33% of the 7–14-year-old children fulfilled diagnostic criteria for PTSD [6]. It is particularly challenging to apply diagnostic criteria during early childhood because of rapid developmental changes and the interdependence of very young children with the primary caregiver [14]. Due to limited self-reflective and verbal abilities, symptoms of children under 6 years are often assessed by parent rating [15, 16]. A correlation between the mothers’ and the children’s symptomatology concerning PTSD [11, 13–15] has been shown as well as an underestimation of PTSD symptom load in parent rating, especially concerning internalized symptoms [16, 17]. These findings underline the need of tools considering child-specific ways of expressing trauma-related stress in addition to parent rating [15]. Across different settings, the pervasiveness of the child’s symptoms may vary, therefore multimodal and observational assessment by multiple informants of the environment, the stressors and the child’s social and emotional functioning is strongly recommended [8, 14].

In previous studies, arousal symptoms were observed as the most frequent trauma-related symptoms in children 7 years and under [16], and furthermore, especially symptoms of hyperarousal are mentioned as a strong predictor of later impairments [17]. As multiple adverse experiences during childhood are associated with aggressive and violent behavior at a later point in time [7] the early evaluation of so-called “survival states “[18] as a marker for loss of behavioral control might be of help in this context.

Within the concept of survival states, the problematic manifestations of traumatic stress are understood both as a result of a child’s emotion and behavior dysregulation, as well as the inadequate capacity of their social environment to provide support. In the framework of Trauma Systems Therapy (TST) [18] there are multiple actors on the child’s needs, chiefly the parents, caregivers and educators. A critical part of TST is the so-called “survival-in-the-moment-states” or “survival states”, defined as “an individual’s experience of the present environment as threatening to his or her survival, with corresponding thoughts, emotions, behaviors, and neurochemical and neurophysiological responses” (p. 46, 18]. Following this thought, the primary developmental pathology of traumatized children is the dysregulation of emotional states when confronted by a stressor [14]. The increased sensitivity to threat cues in the environment might be a reasonable reaction to chronic variable stress caused by living in an unpredictable environment during war, flight and the relocation phase for refugee children [9]. In current descriptions of PTSD diagnostic criteria, corresponding behaviors are not adequately captured [19]. For example, a child might suddenly engage her or his fight response as a result of feeling threatened by a certain cue or wording in the kindergarten. The aggressive behavior, meant to defend herself or himself, is not tolerated and the child is punished. This could lead to repeated experiences of exclusion from social groups and educational disadvantages, hence highlighting the so-called survival states as one of the priority problems of children with a history of trauma exposure [18]. Further, growing up in an unpredictable developmental environment characterized by chronic variable stress is associated with up-regulated diurnal cortisol [9].

The concept of TST and the developmentally modified diagnostic criteria for PTSD in preschool children according to DSM-5 take into account that young children tend to express the core symptoms of PTSD (intrusions, avoidance, alterations in arousal and activity) through behavior (like irritable outbursts, phobic reactions, hyperactivity etc.) rather than being able to communicate cognitive and emotional disturbances [15, 16, 18–20].

Because survival states are rated according to a standardized protocol [18] (see Method) by educators during kindergarten, pre-school or school lessons, this approach adds a complementary perspective to the parents’ view on symptom load and well-being of the potentially traumatized child. In order to answer the question whether survival states might be a useful tool to evaluate PTSD in preschool refugee children, we computed the correlation of survival states with PTSD parent rating, learning performance and biological stress evaluated by salivary cortisol.

In detail the following hypotheses were tested:

H1: The children’s symptom load is closely linked to the parents’ distress level. Children whose parents show higher levels of distress show a higher symptom load in the environment and a higher frequency of survival states in educator rating.

H2: Children with refugee background show a higher frequency of survival states in educator rating than children from an age-matched control group.

H3: Children with higher levels of post-traumatic stress indicated by survival states show a poorer learning performance and display elevated salivary cortisol levels.

## Methods

### Participants

The study was conducted between July 2018 and February 2020. Participants were recruited from several camps for refugees and the Social Pediatric Center (SPZ) at “kboKinderzentrum” in Munich, Germany. Data were collected directly in the camps in separate examination rooms or in the office rooms in the SPZ, respectively. The study included children with refugee background, aged 3 to 7 years. All were attended by at least one parent and were not born in Germany. Each newcomer eligible for our study was contacted by the consultant team and received information about the study. They were asked to participate and invited to the first appointment, with interpreters if necessary. About 5% declined (mostly before informed consent), leaving 72 children and their families willing to participate after informed consent. The control group was recruited in the camps and in the SPZ and consisted of children who were referred due to developmental, language and/or social-emotional problems between April 2019 and February 2020. Children with diagnoses of chronic disabling conditions were excluded.

See Table 1 for description of the sample.

**Table 1:** Demographic and symptom characteristics of the sample

	Refugee Group (***n*** = 52)	Control Group (***n*** = 20)
**Mean age in years** (SD, Range)	5.14 (1.17, 3.00–7.67)	4.99 (1.41, 2.58–6.83)
**Non-verbal IQ** (SD, Range)	79.53 (16.33, 49–118)	86.89 (18.63, 61–133)
**SDQ parent** (SD, Range)	13.93 (6.83, 1–28)	11.71 (5.37, 3–19)
**SDQ educators** (SD, Range)	13.77 (6.90, 0–32)	15.85 (9.19, 4–40)
**CATS trauma-screening**		
• Events reported	32.5 (61.5%)	3 (15%)
• Symptoms (SD, Range)	9.27 (12.17, 0–43)	0
• Children above threshold	15 (29%)	0
• Full DSM-V criteria for PTSD	9 (17%)	0
**Survival States**	28 (54%)	5 (25%)

### Procedure

Informed written consent was given by the parents, and children provided verbal consent. The study protocol was approved by the ethics committee of the Medical Faculty of the Technical University of Munich.

All parents were asked for general information on age, sex, language and cultural background. The parents in the refugee group were asked for the duration of flight and time since arrival in Germany.

While children completed the Scale of Intellectual Functioning (SIF) of the Kaufmann-Assessment-Battery for Children (KABC-II) [21] and the learning task, parents filled out the online questionnaires mentioned below in their native language (“PORTA”) [22]. If parents had difficulties with the written format, the questionnaires were administered as interviews with interpreters, which was the case in the majority of participants.

In the refugee group, saliva samples were collected at home by parents after a practice demonstration in the office and were analyzed using the Demeditec Cortisol free in Saliva ELISA kit at Synlab [23]. The children were advised to spit in small labeled containers in the morning immediately after waking, in the afternoon (between 3 and 4 pm) and in the evening, right before going to bed. Parents were asked to collect samples on two consecutive days. All children with at least three saliva samples composing one daily profile (morning, afternoon and evening) were included in the analysis.

### Assessment

#### Measures

##### Refugee health screener (RHS-15, parent self-rating)

We assessed parental well-being with the “Refugee Health Screener” (RHS-15) [24], by screening for emotional distress as a common marker across psychiatric diagnoses in many ethnic groups. The RHS is an empirically developed standard instrument and we followed the authors’ recommendation that “a score of ≥12 or a distress thermometer score of > 5 is considered a positive case” (p. 6) [24]. We calculated a sum score by multiplying the symptom score with the thermometer score.

##### Child and adolescent trauma screening (CATS, child screening in parent-rating)

All children were screened for potentially traumatic experiences and symptoms of PTSD with the “Child and Adolescent Trauma Screening” (CATS) [25], a short open accessible screening instrument directly based on the DSM-5 criteria for PTSD with satisfactory psychometric properties, constructed with emphasis on sensitivity. We used the recommended cut-off ≥16 (total symptom score) as an indicator of a clinically relevant level of symptoms in preschool children.

##### Strengths and difficulties questionnaire” (SDQ, child assessment in parent- and educator-rating)

The “Strengths and Difficulties Questionnaire” (SDQ) [26] is a fully evaluated and widely used brief behavioral screening questionnaire for the age group 3–17 years with 25 items and forms for parents and educators. The SDQ is reported to be able to distinguish between community and clinical samples [27].

##### TST assessment form (assessment of child’s survival states in educator rating)

We assessed survival states in the environment via educator ratings with the German version of the “TST Assessment Form “[18], translated by Andrea Hahnefeld (2018), asking for “shifts in awareness, affect, and action, when a threat is perceived in the present environment” (p. 399) [18] and also enquiring if these episodes include potentially dangerous behaviors and/or result in any problem with the child’s functioning [18].

##### Kaufmann-assessment-battery for children (KABC-II)

Using the culture-fair, reliable and neuropsychologically developed instrument “Kaufmann-Assessment-Battery for Children “(KABC-II) [21] we assessed the children’s cognitive abilities on a language-free scale (Scale of Intellectual Functioning, SIF). The KABC SIF has good psychometric properties concerning construct validity (.59–.72 with other non-verbal test batteries). Only small score differences between ethnic groups are marked [21].

The memory-task (subtest Atlantis) to assess the children’s short-term learning performance shows low intercorrelations with the general cognitive scale (.28) and is reported to be reliable (.97) and independent from the parents’ migration status [21].

With low correlations with tests for short-term selective attention (−.06 SIF, −.02 Atlantis) both tasks show good discriminant validity [21].

##### Psychometric testing and statistical analyses

We conducted nonparametric correlations (Spearman rank correlation, one-tailed), cross-tabs (refugee vs. control group/survival states yes vs. no) with χ^2^-Test, one-way ANOVA with factors CATS (parent rating) and survival states (educator rating), respectively, and non-parametric Mann-Whitney-U-Test for comparing the salivary cortisol levels in groups with and without survival states in the refugee group. All calculations were done with IBM SPSS Statistics Version 25.

## Results

### Participants

Of initially 72 eligible children with refugee background, complete data could be obtained for 52 children (58% boys, 42% girls) from 44 families, which was mostly due to short-term and unpredictable transfers of the families and personnel turnover in the camps or infrequent participation of the children in playgroups. For 37 refugee children valid cortisol samples could be collected. Participants with and without saliva samples did not differ in age, flight duration, symptom load child and parent, IQ or learning performance. The mean duration of time since arrival in Germany was 19 months (SD 17.72, range 0.5–60 months) after an average flight duration of 24 months (SD 34.70, range 0–140 months). Families were from 14 nations, mostly of Nigerian (14), Afghan (13), Syrian (7) origin in the refugee group, and from 13 nations and mostly of Nigerian (4), German (4) and Turkish (2) origin in the control group. The control group consisted of 20 children born and raised in Germany (45% boys, 55% girls) from 19 families. The groups did not differ in age (t = −.46 (70), *p* > .05), IQ (t = 1.60 (66), *p* > .05) or general symptom load in the SDQ (parent- und educator-rating, t = − 1.22 (66), *p* > .05 and t = 1.04 (70), *p* > .05). See Table 1 for description of the sample.

### Test results

H1: Parents’ own distress level and parent rating of the child vs. children’s symptom load in the environment in educator rating.

Potentially traumatic experiences in parent rating were reported for 32 of the children with refugee background (61,5%) and for 3 children in the control group (15%). No symptoms were reported by the parent rated CATS in the control group, whereas in the refugee group, 15 children (29%) were scored above threshold (see Table 1).

**Fig. 1:**
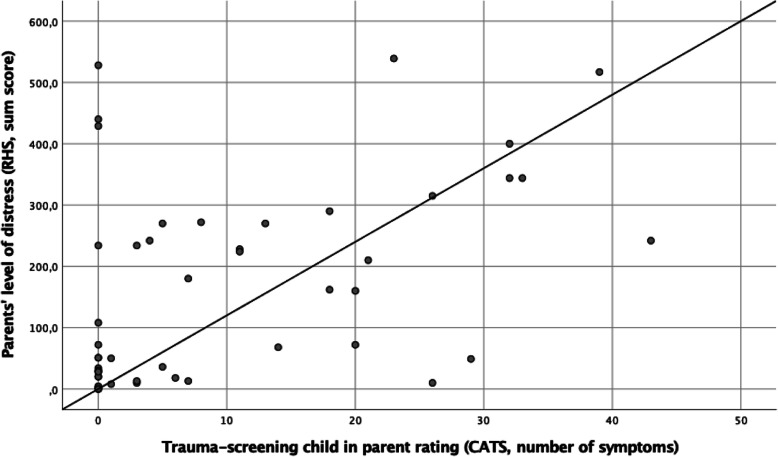
Scatter plot and Spearman correlation coefficient for parent’s level of distress (RHS sum score, self rating) and children’s trauma-related psychopathology (number of symptoms in CATS, parent rating) in the refugee group. *r* = .50, *p* < .001, one-tailed

There were significant intercorrelations within all the variables that were assessed in parent rating. For Spearman rank correlations RHS, CATS, SDQ parent and educator rating see Table 2, for correlation CATS-RHS see Fig. 1.

**Table 2:** Correlation coefficients (Spearman rank correlations, one-tailed) for CATS (parent rating of the child), RHS (parent self-rating), SDQ (parent and educator rating of the child) and survival states (educator rating) in the refugee group

	CATS	RHS	SDQ-parent	SDQ-educator
**CATS**	1	.50^b^	.47^b^	.15
**RHS**		1	.64^b^	.13
**SDQ-parent**			1	.44^b^
**Survival States**	.25^a^	−.03	−.03	.35^b^

^a^*p* < .05

^b^*P* < .001

With correlations of RHS and SDQ educator of *r* = .13 (*p* = .18) and survival states of *r* = −.03 (*p* = .41), the hypothesis that children of highly distressed parents displayed more symptoms in the education environment could not be confirmed.

Concerning the general symptom load (SDQ), parent and educator ratings showed a significant correlation (*r* = .44, *p* < .001), but there was no correlation between CATS in parent rating and general symptom-load in educator rating (*r* = .15, *p* = .14).

H2: Survival states.

Survival states in the education environment were reported for 28 children (54%) in the refugee group and for 5 children (25%) in the control group. χ^2^-Test yielded significant results (χ^2^ = 4,84 (1), *p* < .05), survival states in the education environment were reported significantly more often for refugee children than for control children.

The survival states assessed in educator rating correlated with the CATS-scores above threshold in parent rating (*r* = .25, *p* < .05) and showed a significant correlation with the general symptom score (SDQ) in educator rating (*r* = .35, *p* < .001), see Table 2.

H3: Short-term learning performance and salivary cortisol levels.

**Fig. 2:**
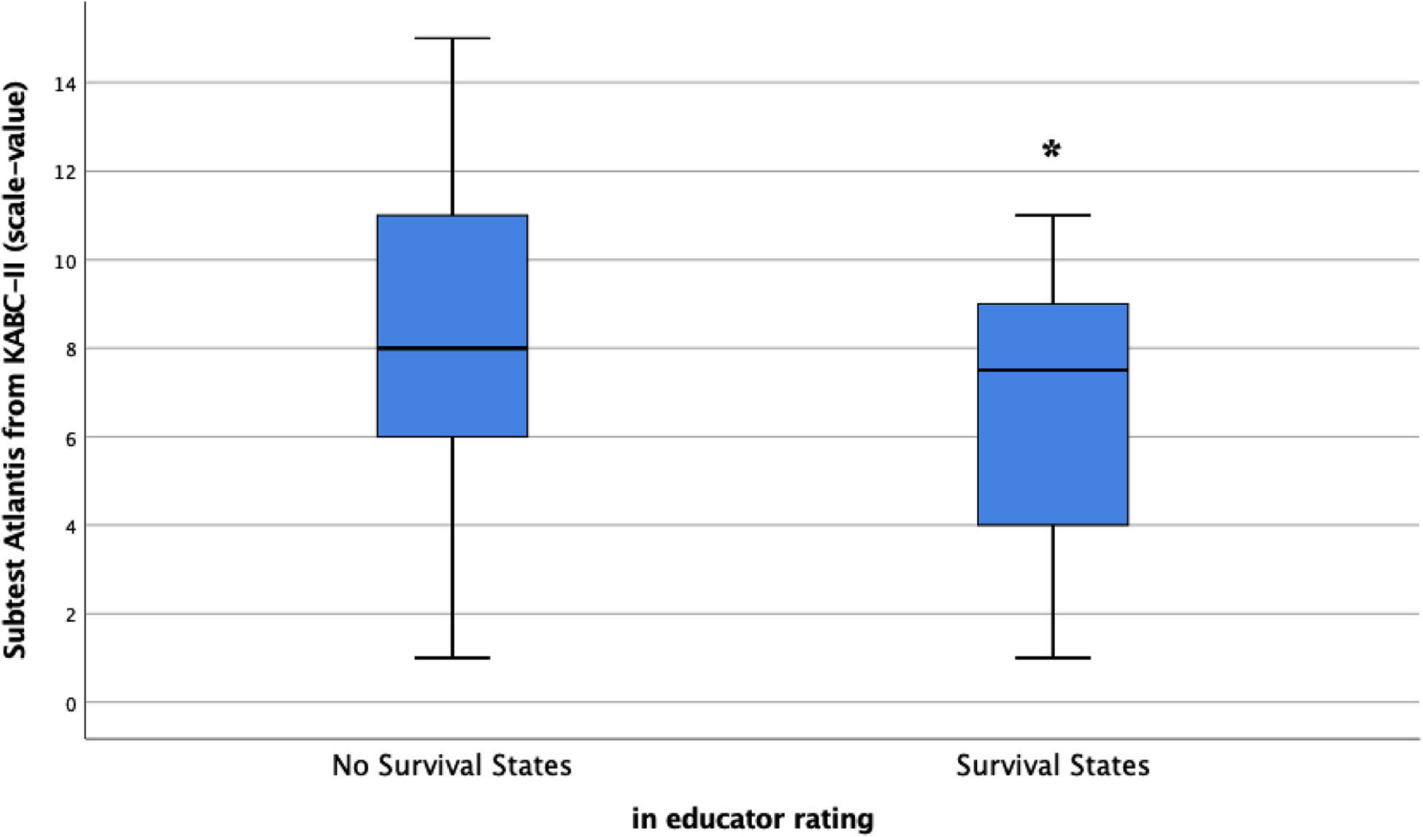
Box-plots and F-value for the refugee group with survival states (rated by educators) as group variable and performance in short-term learning task (in scale values) as dependent variable. *F* = 3.49 (50,1), *p* < .05, one-tailed

In relation to refugee group short-term learning performance, a one-factor ANOVA was performed with IQ and subtest Atlantis as variables. This yielded a significant main effect for subtest Atlantis (*F* = 3.49 (50,1), *p* < .05, one-tailed). Children exhibiting survival states showed lower short-term learning performance (see Fig. 2). There was no significant main effect for IQ (*F* = .46 (47,1), *p* = .25, one-tailed).

By computing ANOVA with group-factor CATS traumatic vs. non-traumatic scoring there were no significant main effects for learning performance or IQ (*F* = 1.25 (50,1), *p* = .13; *F* = 1.22 (47,1), *p* = .14, one-tailed).

**Fig. 3:**
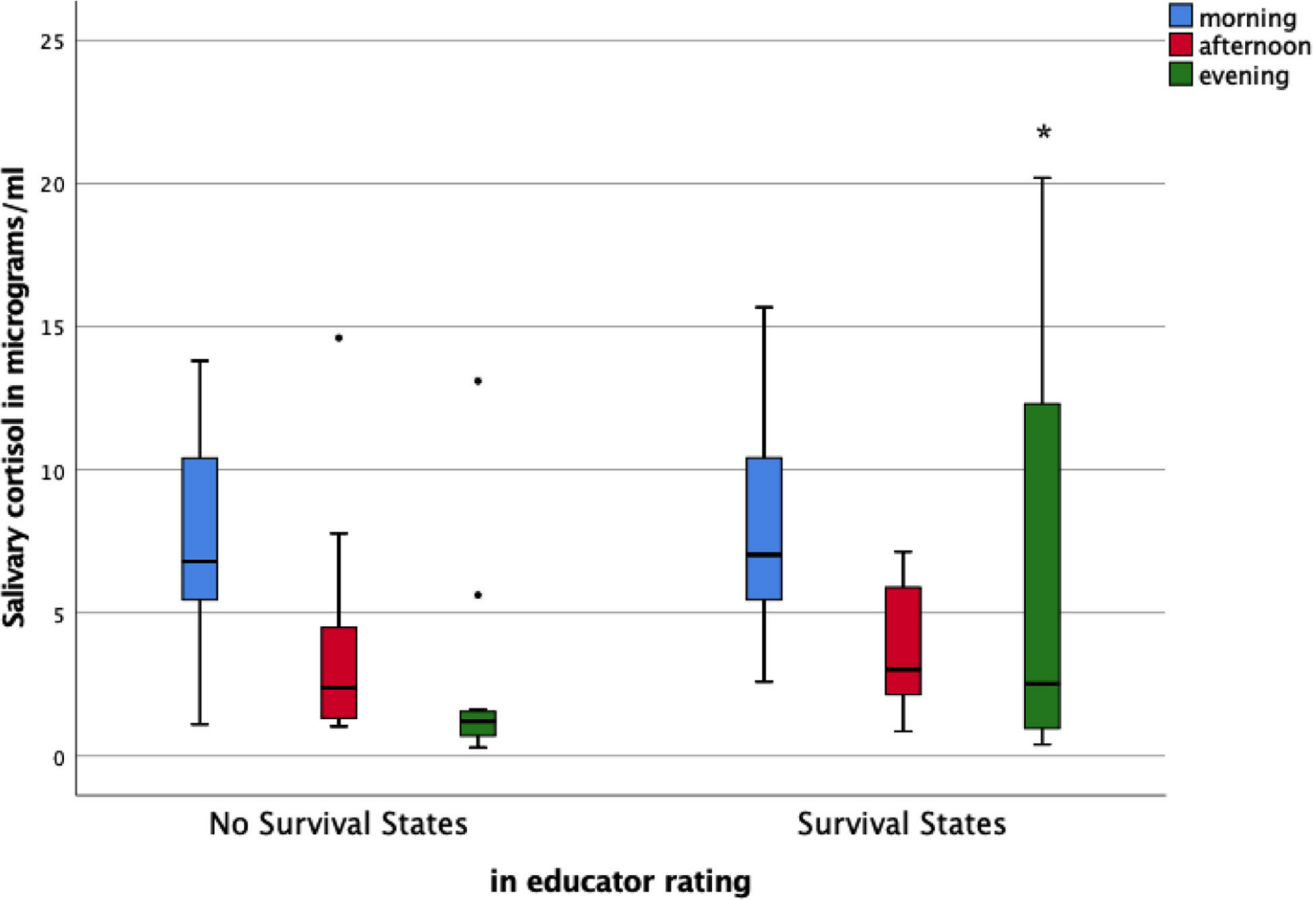
Box-plots and U-values for the refugee group with survival states (rated by educators) as group variable and morning, afternoon and evening values of salivary cortisol (in micrograms/ml) as dependent variables. U = 113, z = − 1.7, *p* < .05, one-tailed, for evening values

In the refugee group, children with survival states displayed significantly higher evening salivary cortisol levels than children without survival states (Mann-Whitney-U_e_ = 113, z = − 1.7, *p* < .05, one-tailed) with a tendency for more variant and higher levels of total diurnal cortisol (Mann-Whitney-U_diurnal_ = 118, z = − 1.5, *p* = .08, one-tailed) and significantly higher levels summed up for afternoon and evening cortisol (Mann-Whitney-U_ae_ = 112, z = − 1.7, *p* < .05, one-tailed), see Fig. 3.

No significant effects could be observed for CATS scores as group variable concerning salivary cortisol level for any time of the day (Mann-Whitney-U_e_ = 128.5, z = −.70, *p* = .25, one-tailed, for evening salivary cortisol, Mann-Whitney-U_diurnal_ = 132, z = −.54, *p* = .28, one-tailed, for diurnal salivary cortisol).

Whereas most of the children without survival states had a normal diurnal slope with elevated levels of cortisol in the morning and declining values during daytime, reaching the nadir at prebedtime in the evening with values close to zero, a number of children in the refugee group with survival states showed elevated levels of cortisol at prebedtime measurement.

As expected, children with survival states showed a weaker short-term learning performance and displayed higher cortisol levels with significant differences in the evening (prebedtime) values than children without survival states, see Figs. 2 and 3.

Children with higher evening and diurnal cortisol levels had longer duration of flight from home country (r_e_ = .45, r_diurnal_ = .56, *p* < .001) and shorter time since arrival in Germany (r_e_ = −.39, r_diurnal_ = −.42, *p* < .05). We also found a longer duration of flight phase for children rated positive in CATS by their parents (*r* = .29, *p* < .05).

## Discussion

To the best of our knowledge, this is the first study evaluating survival states rated by educators as expressions of increased sensitivity to threat cues in the environment in preschool refugee children. Primarily, we confirmed that the refugee children’s symptom load is closely linked to the parents’ distress level, when both is assessed in parent rating. But contrary to our expectations, children with highly distressed parents did not show a higher symptom load or a higher frequency of survival states in the environment assessed by educator rating.

Further, our results yielded that survival states arise more often in refugee children than in control children born and raised in Germany. As a main result concerning survival states within the refugee group, we found poorer learning performance and elevated salivary cortisol levels for those children with survival states in the education environment. There was no corresponding effect for learning performance and salivary cortisol for refugee children rated positive for PTSD in parent rating.

We conclude from this that the assessment of survival states in the environment adds important information to identify the risk for long-term psychiatric disturbances after potentially traumatic experiences in the course of relocation for several reasons: a) documenting highly disruptive behavior expressing elevated arousal in young children, b) focusing on the child in a group environment and c) offering a complementary perspective to the parents’ view on trauma-related child symptomatology by educator rating.

Relating to a) and b) and concerning the role of impairment, adequately regulated social behavior in a group environment can be seen as one of the core competencies during rapid development in childhood. Especially when adapting to a new environment, behavioral regulation is a prerequisite to fulfill further developmental tasks [28]. Language acquisition happens more effectively in social interactions, and the majority of formal education usually takes place in a group environment [29].

However, due to their previous experiences in the face of adversity, some refugee children do still show a behavior which facilitated survival in the face of potential danger but which is highly dysfunctional in the new context of a secure education environment in a non-malevolent ambiance [18, 30]. For example, immediate strong reactions to small triggers signaling danger and self-defending reactions can be life-saving during flight but showing disruptive behavior after being triggered for no obvious reason in the classroom is highly undesirable.

In this context, to offer a complementary perspective to parent rating of the child’s trauma-related symptomatology (c) is particularly important, since solely applying the diagnostic category of PTSD in parent rating is reported to underestimate the children’s trauma-specific symptom load and consecutive impairment [16, 17], especially in the long term [30–35]. In line with that, we found higher rates for survival states in educator rating than CATS-scores above threshold in parent rating in both groups (54% vs. 29% in the refugee group, 25% vs. 0% in the control group). It is remarkable that the children’s general symptom load was not underestimated by their parents as we were able to demonstrate a significant correlation between parent and educator SDQ-rating. This points towards specific reasons for the mismatch concerning their child’s trauma-related psychopathology such as the parents’ own trauma experience and their efforts to deal with its sequela in daily life (for example by reinstituting familiar routines). This might reduce their capacity and judgement of the child’s trauma-related problem load, an assumption which is further supported by our finding that 38.5% of the parents in our highly vulnerable sample of the refugee group did not indicate any potentially traumatic experiences for their children after flight and relocation. Perhaps parents were reluctant in reporting these experiences for their children in a questionnaire format. Communicating their child’s traumatic experiences could as well be difficult owing to the parents’ own PTSD symptoms (e.g. avoidance, concentration problems or strong emotional arousal) [8]. Correspondingly, in a prospective follow-up of preschool children by Scheeringa et al. [32] two to three times more children were considered functionally impaired by their caregivers than were diagnosed with PTSD in parent rating, with growing discrepancy between these two measures in the course of 2 years. The authors found that overall PTSD symptoms (evaluated by parent interviews) did not decrease over time, even with community treatment. They report significantly more children to be functionally impaired one (49%) and 2 years (74%) after first assessment for PTSD (23%). The number of positive ratings for survival states in our study rather equals the rate of children being functionally impaired 1 year after first assessment for PTSD in the study by Scheeringa et al. [32]. Because we and others [8, 16, 33] provide evidence that trauma-specific aspects of their child’s mental health may be difficult to be perceived or communicated by parents, educator rating of survival states might be an important supplement in PTSD diagnosis. Considering this, we correlated survival states with other markers that are connected with high levels of stress and impairment.

It is well known that early adversity and its impact on later functioning is associated with alterations in the Hypothalamic-Pituitary-Adrenal (HPA) axis [9, 30, 36–39] with initial upregulation of the stress response system (hypercortisolism) and later downregulation [40] in a way that early life stress programs the developing brain to react differently to future stressors [37, 41, 42]. In our study, we found refugee children with survival states to display higher levels of salivary cortisol in the evening than those children who did not show that kind of episodes in the education environment. As illustrated in Fig. 3, cortisol was high near wake-up for both groups and decreased over the day. The group without survival states showed a normal diurnal rhythm with declines of pre-bedtime values of cortisol close to zero. For children with survival states there is a marked inability to bring cortisol concentrations to low levels in the evening, which might reflect a fundamental dysregulation of the neuroendocrine system, as reported in previous studies [36, 38, 43]. In their review, Kuhlman et al. [9] conclude that children growing up in conditions of unpredictable events show increased neural sensitivity to threat which is in turn related to more frequent activation of the physiological stress system and elevated diurnal cortisol. Comparably, we found higher cortisol levels in children with experiences of war/flight/relocation not only to be correlated with survival states but also with longer flight durations and shorter time of residence in Germany. As the measurement point for salivary cortisol in our sample was relatively close to the traumatic experiences or even during ongoing stress, we may have captured the initial up-regulation of the hypothalamic-pituitary-adrenocortical (HPA) system [40].

Furthermore, there is a line of evidence linking higher levels of daytime cortisol in combination with adversity and high arousal symptoms to poorer cognitive development in the long term [38, 44, 45]. Raffington et al. [46] performed a study concerning the link between cortisol secretion, reduced hippocampal volume and memory performance in children from high stress environments. They found lower family income to be associated with smaller hippocampal volume, flatter cortisol awakening reaction and hyporeactivity in cortisol stress reaction. This, in turn, was related to lower memory function [46]. An early impairment of learning performance in children with high levels of salivary cortisol might be setting the stage for poorer cognitive outcome in the long-term. This is especially important as the majority of the children in our sample were examined during the sensitive phase of hippocampus development at age 3–5 years [36, 45] and those with disturbing survival states displayed a lower short-term learning performance.

Although the one-point survey-design of the study does not allow causal inferences with our data, we established the link between survival states as behavioral disturbances due to high arousal, elevated cortisol levels in the evening and learning difficulties.

While using widespread standardized questionnaires, we noticed that parents had trouble with the forms and were sometimes restrained to answer or disclose too much. Other studies report similar issues, suggesting the validity of psycho-diagnostic measures developed in western populations might be restrictive for refugees from non-western countries [10]. As it is common practice for other psychiatric disorders during childhood (for example, autism spectrum disorder and attention deficit and hyperactivity disorder are characterized by certain patterns of behavior across multiple settings) [47], we added a complementary perspective to parent view with supplemental assessment of survival states by educators in a different context. Even more so, shifting the focus from the individual or families to a more public health approach as it is done in global mental health might be useful in our system as well when being confronted with patients from different cultural backgrounds [48]. A suggestion for further research might be to consider the effects of corresponding therapeutic treatment according to trauma systems therapy (TST) [18] for children with high biological stress and associated learning difficulties: Concluding from our results, treatment of dysregulated young (refugee) children should not only focus on child and family, but also operate through trauma-sensitive child-care in the social and education environment.

The unique combination of the multi-rater- and multi-method-perspective to link behavioral and questionnaire data with learning performance and the objectively observable underlying neurobiological marker of salivary cortisol can be seen as a strength of this study, measuring not only the occurrence of events but also their impact.

For organizational reasons and because of characteristics of the refugee sample, we could not control for socioeconomic status. Voluntariness and the particular challenge for the parents to collect saliva samples add further potential bias of the sample, reducing the ability to extrapolate the findings, although we did not find any differences in the variables assessed between children with and without saliva samples.

The heterogeneity of the sample with refugees from 14 different countries limits the generalization for certain ethnic groups, but at the same time yields a good representation of the general refugee population in Germany. Chiefly underlying deficits in behavior regulation, salivary cortisol levels and learning behavior might be universal and not limited to certain ethnic subgroups, as in Scheeringa and Zeanah’s study [32] differences by ethnic background and gender were reported largely non-significant.

Additionally, the control group was also composed of families with a multinational background. As a clinical sample, the control group is from a help-seeking cohort and not representing the general population, which further highlights the relevance of our results.

## Conclusions

In our refugee sample, survival states in the education environment were reported more often than trauma-screenings above threshold in parent rating. The assessment of survival states proved to be feasible and valid, as we found significant correlations with lower learning performance and elevated evening salivary cortisol levels, which in turn are known to be associated with adversity and higher levels of distress. As for other psychiatric disorders during childhood, the educator perspective should be considered as an additional measure for the assessment of PTSD symptoms in preschool refugee children.

## Data Availability

The datasets used and analyzed during the current study are available from the corresponding author on reasonable request.

## Abbreviations

PTSD: Posttraumatic Stress Disorder
CATS: Child and Adolescent Trauma Screening
RHS: Refugee Health Screener
SDQ: Strengths and Difficulties Questionnaire
KABC-II: Kaufmann Assessment Battery for Children
TST: Trauma Systems Therapy
SPZ: Social Pediatric Center
SIF: Scale of Intellectual Functioning (KABC-II)
PORTA: Providing Online Ressource and Trauma Assessment for Refugees
HPA: Hypothalamic-Pituitary-Adrenal
